# Comparison of [^18^F]fluciclovine and [^18^F]FDG PET/CT in Newly Diagnosed Multiple Myeloma Patients

**DOI:** 10.1007/s11307-022-01734-0

**Published:** 2022-05-02

**Authors:** Caroline Stokke, Jakob Nordberg Nørgaard, Hilde Feiring Phillips, Alexander Sherwani, Syed Nuruddin, James Connelly, Fredrik Schjesvold, Mona-Elisabeth Revheim

**Affiliations:** 1 Division for Radiology and Nuclear Medicine, Oslo University Hospital, Oslo, Norway; 2 Dep. of Physics, University of Oslo, Oslo, Norway; 3 Oslo Myeloma Center, Oslo University Hospital, Oslo, Norway; 4 Institute of Clinical Medicine, University of Oslo, Oslo, Norway; 5 Norwegian Medical Cyclotron Centre, Oslo, Norway; 6 KG Jebsen Center for B Cell Malignancies, University of Oslo, Oslo, Norway

**Keywords:** Multiple myeloma, [^18^F]fluciclovine, [^18^F]FACBC, Axumin, PET/CT, [^18^F]FDG, Anti-1-amino-3-[^18^F]-fluorocyclobutane-1-carboxylic acid

## Abstract

**Purpose:**

[^18^F]FDG PET/CT in multiple myeloma (MM) is currently the best technology to demonstrate patchy and extramedullary disease. However, [^18^F]FDG PET has some limitations, and imaging with alternative tracers should be explored. In this study, we aimed to evaluate the performance of [^18^F]fluciclovine PET compared to [^18^F]FDG PET in newly diagnosed MM patients.

**Procedures:**

Thirteen newly diagnosed transplant eligible MM patients were imaged both with [^18^F]FDG PET/CT and [^18^F]fluciclovine PET/CT within 1 week in a prospective study. The subjects were visually assessed positive or negative for disease. The number of lesions and the SUV_max_ of selected lesions were measured for both tracers. Furthermore, tracer uptake ratios were obtained by dividing lesion SUV_max_ by blood or bone marrow SUV_max_. Between-group differences and correlations were assessed with paired *t*-tests and Pearson tests. Bone marrow SUVs were compared to bone marrow plasma cell percentage in biopsy samples.

**Results:**

Nine subjects were assessed positively by [^18^F]FDG PET (69%) and 12 positives by [^18^F]fluciclovine PET (92%). All positive subjects had [^18^F]fluciclovine scans that were qualitatively scored as easier to interpret visually than the [^18^F]FDG scans. The number of lesions was also higher; seven of nine subjects with distinct hot spots on [^18^F]fluciclovine PET had fewer or no visible lesions on [^18^F]FDG PET. The mean lesion SUV_max_ values were 8.2 and 3.8 for [^18^F]fluciclovine and [^18^F]FDG, respectively. The mean tumour to blood values were 6.4 and 2.0 for [^18^F]fluciclovine and [^18^F]FDG, and the mean ratios between tumour and bone marrow were 2.1 and 1.5 for [^18^F]fluciclovine and [^18^F]FDG. The lesion SUV_max_ and ratios were significantly higher for [^18^F]fluciclovine (all *p* < 0.01).

Local [^18^F]fluciclovine SUV_max_ or SUV_mean_ values in os ilium and the percentage of plasma cells in bone marrow biopsies were linearly correlated (*p* = 0.048). There were no significant correlations between [^18^F]FDG SUVs and plasma cells (*p* = 0.82).

**Conclusions:**

Based on this pilot study, [^18^F]fluciclovine is a promising tracer for MM. The visual and semi-quantitative evaluations indicate that [^18^F]fluciclovine PET/CT can out-perform [^18^F]FDG PET/CT at diagnosis.

**Supplementary Information:**

The online version contains supplementary material available at 10.1007/s11307-022-01734-0.

## Introduction

The detection of minimal residual disease (MRD) has become increasingly important with the introduction of more effective treatment options for multiple myeloma (MM). Flow cytometry and next-generation sequencing, two different cell-sampling-based methods for detecting MRD in MM, are currently in use (1). However, given that about 80% of MM patients suffer from patchy manifestations of the disease (2), it is advantageous to include methods for whole-body assessments. PET imaging using 2-Deoxy-2-[^18^F]-fluoroglucose ([^18^F]FDG) is by The International Myeloma Working Group considered the best technology to demonstrate patchy and extramedullary disease in MM (3). [^18^F]FDG PET has been shown to predict outcomes in MM (4–6), and it may be of special importance in response evaluation (7). [^18^F]FDG PET negativity after treatment has been demonstrated as an independent predictor of prolonged survival for patients with conventionally defined complete response (5, 8–10).

However, [^18^F]FDG PET has some limitations, such as that 8–10% of MM patients are [^18^F]FDG negative at diagnosis (3, 10, 11). [^18^F]FDG can also accumulate in areas of reparative inflammation and therefore contribute to false-positive assessments. Other PET tracers may therefore be of significant value. Previously studies have included for example the CXCR4-targeting [^68^ Ga]Ga-pentixafor (12, 13), [^11^C]acetate *(14)*, Na[^18^F]F (9, 15), the CD38-targeted [^64^Cu]- or [^89^Zr]-DFO-daratumumab (16, 17), and carbon-11- or fluorine-18 labeled choline (18–20). Also, the amino acid-based tracer [^11^C]methionine is a promising non-[^18^F]FDG tracer for MM, and it has shown higher sensitivity in comparison to [^18^F]FDG to detect intra- and extramedullary MM (21, 22). Unfortunately, the short half-life of carbon-11 (20 min) renders this tracer inconvenient for most centers. Anti-1-amino-3-[^18^F]-fluorocyclobutane-1 -carboxylic acid ([^18^F]fluciclovine) is an amino acid-based PET tracer analogous to leucine with a half-life of 110 min (23). It has demonstrated similar uptake patterns to [^11^C]methionine (24) and is approved by the Food and Drug Administration for detection of prostate cancer recurrence in patients with elevated PSA levels. In a recent study for patients with prostate cancer, [^18^F]fluciclovine first identified an incidental second primary neoplasm in 2.7% of the patients (25). The aim of the current study was to investigate the performance of [^18^F]fluciclovine for MM patients, primarily by visual and semi-quantitative comparisons with [^18^F]FDG PET but also by the correlation between biopsy results and bone marrow standardised uptake values (SUVs).

## Material and Methods

### Study Design and Patient Characteristics

MM patients suitable for autologous stem cell transplantation (ASCT) treatment, of age 18 years and older, were eligible for inclusion in this prospective study. Fourteen newly diagnosed MM patients were screened and included in the trial; there were no screen failures. One of the fourteen patients originally included (number 06) withdrew before imaging, therefore thirteen patients were assessed both with [^18^F]FDG PET/CT and [^18^F]fluciclovine PET/CT. Age, sex, and disease risk factors of all subjects were recorded and are described in Table 1. In addition to imaging at diagnosis, both PET examinations were repeated 3 months after ASCT. The study was approved by the National Ethics Committee, and all subjects signed an informed consent form. The ClinicalTrials.gov Identifier is NCT03966443.

**Table 1:** Patient characteristics

Subject	Age (y)	Sex	Lytic lesions (yes/no)	Hemoglobin (g/dl)	eGFR (CKD-EPI, ml/min/1.73m2)	ISS	R-ISS	Calcium uncorrected/free	Lactate dehydrogenase (U/L)	Biopsy Crista (% plasma cells)	High risk cytogenetics
01	54	M	y	14.0	98	1	1	2.32/1.26	163	10	Negative
02	71	F	y	14.0	98	1	N.A	2.34/1.20	231	10–12	N.A
03	47	F	y	10.7	102	2	2	2.21/1.22	134	N.A. (aspirate sternum: 45%)	Negative
04	46	M	n	14.7	110	1	1	2.37/N.A	147	10	Negative
05	74	M	n	13.1	69	1	1	2.34/1.25	151	30	Negative
07	59	F	y	13.1	106	1	1	2.35/N.A	140	N.A	Negative
08	77	M	n	11.2	69	1	1	2.22/1.17	138	40–50	Yes. t(14;20), 13q-
09	55	F	y	9.8	60	2	2	2.34/1.25	106	80	Negative
10	52	M	n	12.8	76	1	1	2.25/1.20	108	30	Negative
11	47	M	y	9.1	59	3	2	2.84/1.46	173	N.A. (aspirate sternum: 40–50%)	Negative
12	68	M	y	12.9	82	2	2	2.61/1.35	109	60	Negative
13	69	M	n	10.7	88	1	1	2.40/1.26	150	20	Yes. t(14;20), 1q +
14	60	M	y	14.0	65	1	2	2.18/1.18	237	40–45	Negative

### Radiopharmaceutical Administration

[^18^F]FDG and [^18^F]fluciclovine were produced on the automated synthesis module FASTlab™ 1 (GE Healthcare) in accordance with good manufacturing practices. The patients fasted for 6 h prior to injection of 4 MBq/kg [^18^F]fluciclovine (range 259–435 MBq). For the [^18^F]FDG examinations, the patients fasted for 6 h, and 3 MBq/kg [^18^F]FDG was injected (range 188–312 MBq). The absorbed doses from the tracer administrations for an 80 kg patient are estimated to 3.4 and 4.6 mSv from [^18^F]fluciclovine and [^18^F]FDG, respectively (26, 27).

### Image Acquisition and Reconstruction

All scans were performed on the same Discovery MI (GE Healthcare) PET/CT scanner. To establish optimum scanning parameters, [^18^F]fluciclovine PET imaging was initially repeated at several time points, approximately 30, 60, and/or 120 min post injection (p.i.) in the first three subjects with 1- to 2.5 min acquisition per bed position. For subsequent subjects, whole body (WB) acquisitions of 1 min per bed position after 15 min p.i. were performed. A low-dose CT scan without contrast enhancement was performed for attenuation correction and anatomic information before the PET acquisition (120 kV, noise index 34.5). PET data were reconstructed using VP FX Q.Clear 400 with a matrix of 384, plus VP FX SharpIR, std filter 5, 16/3 subsets/iterations, and a matrix of 256. The latter reconstruction was used for SUV measurements. The reconstructed slice thickness was 2.79 mm.

For all but one of the patients (subject 08), the [^18^F]FDG PET/CT and the [^18^F]fluciclovine PET/CT were performed within 1 week of each other in random order. The [^18^F]FDG PET was acquired approximately 60 min p.i. (range 54–88) with 2.5 min acquisition per bed position. A low dose CT was performed, using the same parameters as for the [^18^F]fluciclovine PET/CT examinations. The PET reconstruction parameters were identical to those of the [^18^F]fluciclovine PET.

### Image Analysis

Visual assessments were performed by two nuclear medicine physicians independently, and the final evaluation of positivity or negativity was reached in consensus. The [^18^F]fluciclovine and [^18^F]FDG examinations were assessed in random order. For [^18^F]FDG PET/CT scans, the positive/negative assessment was performed based on presence of active lesions and/or high uptake in bone marrow. The IMPeTUs criteria were used as guidance (28). Also for the [^18^F]fluciclovine PET/CT examinations was the overall assessment based on presence of active lesions and/or the appearance of bone marrow uptake (defined as diffuse uptake above physiological uptake). In addition, for positive scans, the images were later qualitatively scored as more, equally or less, easy to interpret compared to the [^18^F]FDG images.

The approximate numbers of active lesions (areas of visually increased tracer uptake compared to background) were categorised as 0, 1, 2, 3, 4, 5, 6–10, 11–20, 21–50, 51–100 or > 100 lesions by an experienced nuclear medicine specialist. SUVs were measured using 1 cm^3^ volumes of interest in liver (SUV_mean_), mediastinal blood (SUV_max_), and os ilium (SUV_max_ and SUV_mean_). The bone marrow regions were placed in areas avoiding focal hot spots, displaying as homogeneous uptake as possible. Also, for all subjects with active lesions, SUV_max_ values were measured for three of the lesions demonstrating the highest uptake on the [^18^F]fluciclovine PET examination and the same locations on the [^18^F]FDG PET examination. Similarly, three of the lesions demonstrating the highest uptake on the [^18^F]FDG PET examinations were also selected, and SUVs were measured from both image sets. Measurements for a total of up to six lesions were hence obtained for each subject. The measured locations were matched between the image sets in a rigorously manner by an experienced nuclear medicine physician. Tumour-to-normal tissue ratios were obtained by dividing lesion SUV_max_ by blood or bone marrow SUV_max_. For the first three subjects with imaging at later time points, the first time point was used to measure SUVs. Syngo.via VB30 software (Siemens Healthineers) was used for the measurements.

### Statistics

Paired *t*-tests and Pearson correlation tests were performed to investigate relationships between tumour SUVs and between tumour to blood or bone marrow ratios for [^18^F]fluciclovine PET and [^18^F]FDG PET. Pearson correlation tests were also used to investigate the correlation between the percentage of plasma cells and SUVs in the bone marrow. The first three subjects were excluded from all analyses as their [^18^F]fluciclovine PETs were obtained at later time points than the rest. IBM SPSS version 25 was used for the statistical analyses.

## Results

### Visual Assessments

**Fig. 1.**
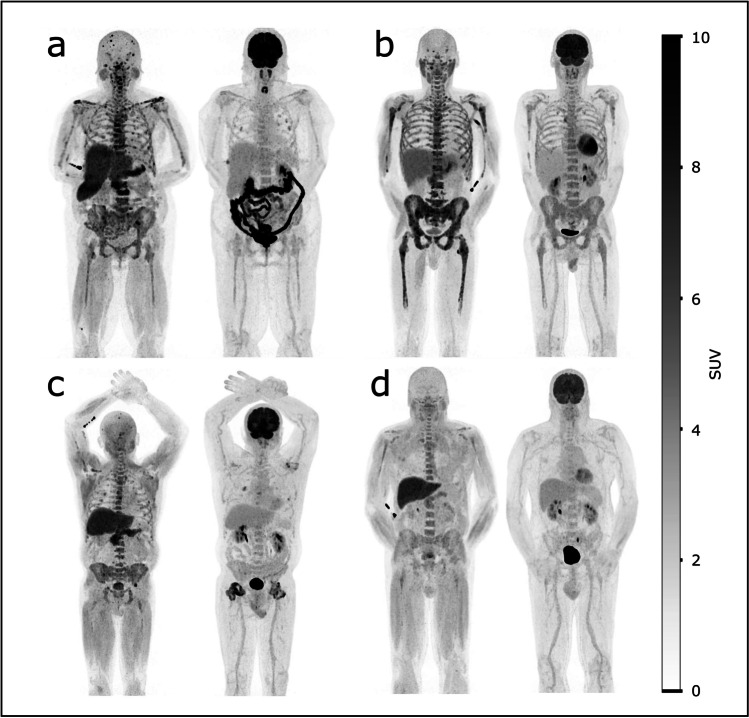
Examples of [^18^F]fluciclovine and [^18^F]FDG PET images. **a** Left panel: [^18^F]fluciclovine PET maximum intensity projection (MIP) of a 59 years old female (subject 07), acquired 15 min p.i. Right panel: [^18^F]FDG PET MIP of the same subject. The subject demonstrated multiple distinct uptakes of [^18^F]fluciclovine, while the [^18^F]FDG PET examination only showed a few weakly positive focal uptakes. **b** [^18^F]fluciclovine and [^18^F]FDG image sets of subject 11. **c** [^18^F]fluciclovine and [^18^F]FDG image sets of subject 12. **d** [^18^F]fluciclovine and [^18^F]FDG image sets of subject 05. Subjects 07, 11, and 12 were positive on both examinations, while 05 was negative on [^18^F]FDG PET and positive on [^18^F]fluciclovine. The [^18^F]fluciclovine and [^18^F]FDG examinations were performed within 48 h for all displayed subjects. The intensity scale is identical for all images, indicated by the bar.

Of the 13 subjects included, nine were assessed positive by [^18^F]FDG PET (69%) and 12 positive by [^18^F]fluciclovine PET (92%) (Table 2). For [^18^F]fluciclovine, the readers’ independent assessments were in agreement for all subjects. Examples of representative patients are shown in Fig. 1. For the 12 subjects showing positivity on [^18^F]fluciclovine PET, the scans were all assessed as easier to interpret than the corresponding [^18^F]FDG PET scans (Table 2). Typically, this was based on both bone marrow appearance and the number and interpretability of active lesions, but for three subjects, it was primarily based on bone marrow contribution (subjects 04, 10, and 13), and one subject with a single lesion with soft-tissue involvement in os frontalis had more clearly distinguishable uptake of [^18^F]fluciclovine than of [^18^F]FDG in that lesion (subject 01). PET images of all subjects with [^18^F]Fluciclovine scan acquired 15 min p.i. are shown in Supplementary Fig. 1.

**Table 2:** Imaging and visual PET assessments

Subject	[^18^F]fluciclovine imaging time points (min p.i.)	[^18^F]fluciclovine assessment (positive/negative)	[^18^F]FDG assessment (positive/negative)	Number of lesions with increased uptake	
				[^18^F]fluciclovine PET	[^18^F]FDG PET
01	61	p	p	1	1
02	32, 60, 120	p	p	6–10	6–10
03	39	p	p	21–50	11–20
04	15	p	n	N.A	N.A
05	15	p	n	6–10	N.A
07	15	p	p	> 100	21–50
08	15	n	n	N.A	N.A
09	15	p	p	21–50	N.A
10	15	p	n	N.A	N.A
11	15	p	p	> 100	21–50
12	15	p	p	> 100	6–10
13	15	p	p	N.A	N.A
14	15	p	p	> 100	3

For seven of the nine subjects (78%) showing focal uptake on [^18^F]fluciclovine PET, the number of hotspots was higher than on [^18^F]FDG PET. Two subjects had approximately the same number of distinguishable lesions.

### Acquisition Protocol

**Fig. 2.**
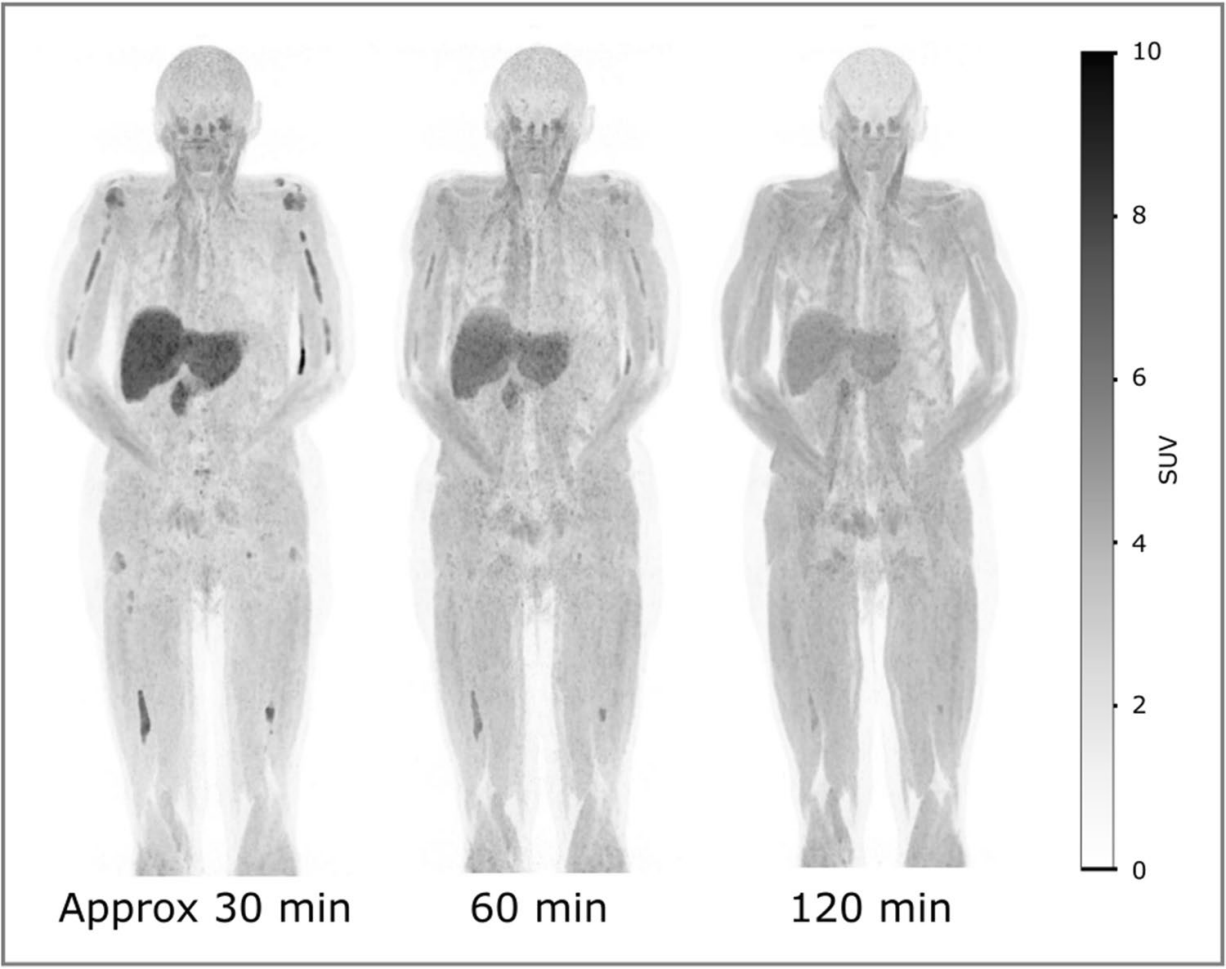
[^18^F]fluciclovine PET MIP images obtained at various time points p.i. of one of the first two subjects included (subject 02). The same intensity scale is used for all images. Already after 30 min, much of the tracer can be seen to have accumulated in muscle tissue, and acquisitions of later subjects were therefore initiated at 15 min p.i

The first three subjects (01–03) were imaged at one or multiple time points ranging from approximately 30–120 min p.i. (Table 2). Rapid washout from lesions was observed, and pronounced uptake in muscle was also found, increasing with time (Fig. 2). Therefore, WB imaging of later subjects was initiated already at 15 min p.i.. This avoided background accumulation in muscles.

### Semi-Quantitative Measurements

**Fig. 3.**
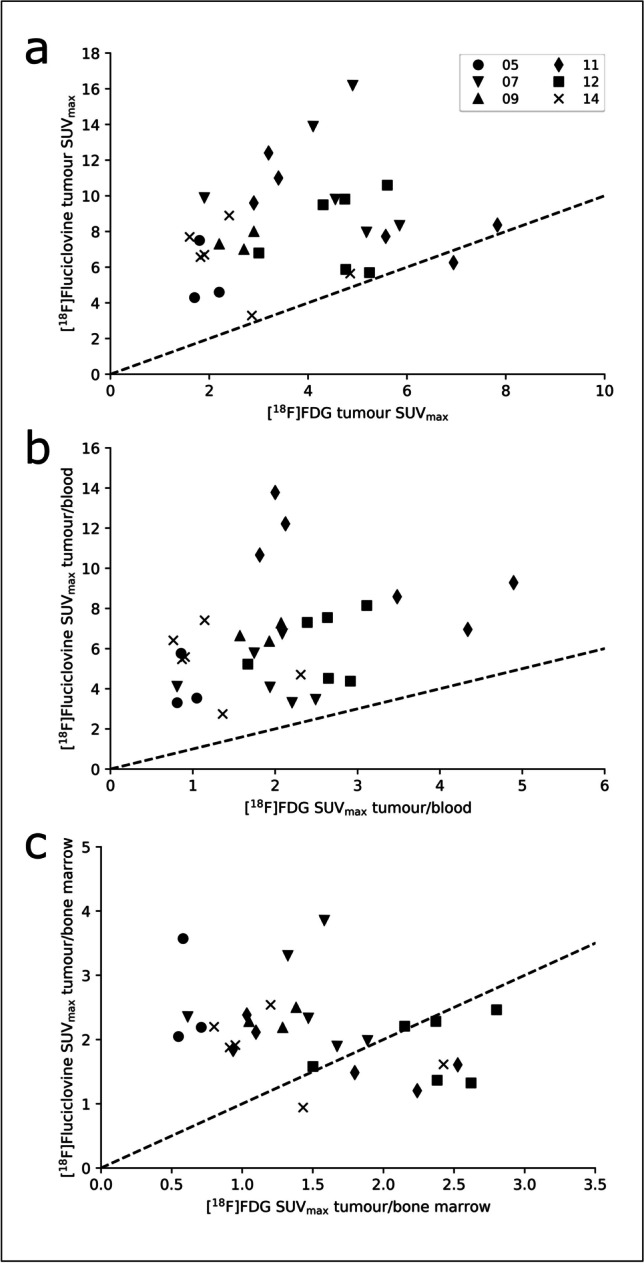
**a** Scatterplot of SUV_max_ for selected lesions with high uptake after imaging with [^18^F]fluciclovine and [^18^F]FDG PET. In this figure, only subjects imaged at 15 min p.i. of [^18^F]fluciclovine are included (subject 04–14). The unity line (dotted line) is also included to indicate the differences in absolute values. **b** Scatterplot of the ratio between tumour SUV_max_ and blood SUV_max_ in subjects imaged with [^18^F]fluciclovine and [^18^F]FDG PET. **c** Scatterplot of the ratio between tumour SUV_max_ and bone marrow SUV_max_ in patients imaged with [^18^F]fluciclovine and [^18^F]FDG PET. The same lesions are included in panels **b** and **c** as in panel **a**. Significant differences (all *p* < 0.01) were found for all three data sets.

Including only subjects who underwent [^18^F]fluciclovine imaging 15 min p.i., the mean SUV_max_ value for lesions were 8.2 (range 3.3–16.2) and 3.8 (range 1.6–7.8) for [^18^F]fluciclovine PET and [^18^F]FDG PET, respectively. The according mean tumour to blood SUV_max_ values were 6.4 (range 2.8–13.8) and 2.0 (range 0.8–4.9), and the mean ratios between tumour and bone marrow SUV_max_ were 2.1 (range 0.9–3.9) and 1.5 (range 0.5–2.8) for [^18^F]fluciclovine and [^18^F]FDG PET, respectively. The three paired sample *t*-tests between tumour SUV_max_, tumour to blood ratio, and tumour to bone marrow ratio for the two tracers all showed significant differences (all *p* < 0,01). The ratios of tumour SUV_max_ to liver SUV_mean_ were approximately identical; 1.3 (range 0.5–3.5) for [^18^F]fluciclovine and 1.2 (range 0.4–3.5)for [^18^F]FDG. The three subjects that were imaged at later time points are excluded from statistical analyses involving tumour SUVs (and from Fig. 3), but measurements for all subjects are shown in Table 3. Pearson tests showed no linear correlations for tumour SUV_max_, the ratio of tumour/blood or the ratio of tumour/bone marrow for [^18^F]fluciclovine vs [^18^F]FDG (*p*-values 0.32, 0.09, and 0.7, respectively).

**Table 3:** SUVs for [^18^F]FDG and [^18^F]fluciclovine PET exams

Subject	Lesion SUV_max_ Patient average for selected lesions (range)		SUV_max_ blood		SUV_mean_ liver		SUV_max_/SUV_mean_ bone marrow	
	[^18^F]fluciclovine PET*	[^18^F]FDG PET*	[^18^F]fluciclovine PET	[^18^F]FDG PET	[^18^F]fluciclovine PET	[^18^F]FDG PET	[^18^F]fluciclovine PET	[^18^F]FDG PET
01^†^	4.1 (4.1–4.1)	3.0 (3.0–3.0)	1.3	2.0	4.1	2.1	1.8/1.3	2.5/1.7
02^†^	6.0 (5.4–6.9)	4.2 (3.7–4.5)	1.2	2.5	5.5	2.4	1.6/1.1	1.5/1.0
03^†^	5.3 (3.9–5.8)	3.9 (3.2–4.9)	1.3	2.3	5.3	2.4	2.8/2.2	2.9/2.0
04	N.A	N.A	-	-	-	-	1.9/1.7	2.6/1.7
05	5.5 (4.3–7.5)	1.9 (1.7–2.2)	1.3	2.1	5.9	2.3	2.1/1.5	3.1/1.7
07	11.0 (8.0–16.2)	4.4 (1.9–5.9)	2.4	2.4	5.6	2.3	4.2/3.9	3.1/2.2
08	N.A	N.A	-	-	-	-	2.5/2.2	3.5/2.5
09	7.4 (7.0–8.0)	2.6 (2.2–2.9)	1.1	1.4	4.4	1.5	3.2/2.2	2.1/1.4
10	N.A	N.A	-	-	-	-	1.3/1.0	0.6/0.5
11	9.2 (6.3–12.4)	5.0 (2.9–7.8)	0.9	1.6	3.9	1.4	5.2/4.6	2.2/1.4
12	8.1 (5.7–10.6)	4.6 (3.0–5.6)	1.3	1.8	6.1	1.7	4.3/2.7	2.0/1.5
13	N.A	N.A	-	-	-	-	2.0/1.6	1.1/0.6
14	6.5 (3.3–8.9)	2.6 (1.6–4.8)	1.2	2.1	5.9	2.1	3.5/2.0	2.0/1.4

^†^Note that the first subjects were scanned at later time points p.i. and the [^18^F]fluciclovine SUVs will not necessarily be comparable to the others. * The same locations were used to measure SUVs at both PET examinations, even if they only had a qualitatively distinguishable focus of either [^18^F]FDG PET or [^18^F]fluciclovine PET. Therefore, subject 05 and 09—who have no [^18^F]FDG avid lesions indicated in Table 2- are still assigned a value here

### Bone Marrow Biopsy and PET Results

**Fig. 4.**
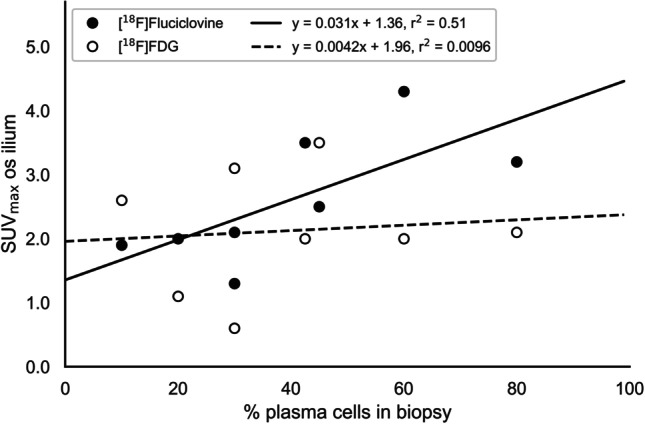
SUV_max_ measured in reference bone marrow site (os ilium) plotted against the percentage of malignant plasma cells in local crista biopsies. Only subjects imaged at 15 min p.i. of [^18^F]fluciclovine, with crista biopsies, are included (subjects 04–14, excluding 07 and 11). The [^18^F]fluciclovine regression line is displayed as solid and the [^18^F]FDG line as dotted. There was a significant correlation between [^18^F]fluciclovine values and the percentage of plasma cells (*p* = 0.048), but not between [^18^F]FDG values and the percentage of cells (*p* = 0.82).

Based on the eight patients with available biopsy results from crista iliaca and [^18^F]fluciclovine imaging performed at 15 min p.i., there was a linear significant correlation between local [^18^F]fluciclovine SUV_max_ in os ilium and the percentage of plasma cells in bone marrow (*p* = 0.048; Fig. 4). There were no significant correlation between [^18^F]FDG SUV and the percentage of plasma cells (*p* = 0.82 for SUV_max_).

## Discussion

In this study, we have evaluated the visual and semi-quantitative performance of [^18^F]fluciclovine PET compared to [^18^F]FDG PET for newly diagnosed MM patients. Although the study size is limited (*N* = 13), [^18^F]fluciclovine PET appears to outperform [^18^F]FDG PET, with a higher number of subjects characterised as positive (92 vs 69%, respectively). Accordingly, higher tumour SUVs and tumour to normal tissue ratios were also observed.

Through investigations of imaging at different time points, we found acquisitions performed rapidly after injection of [^18^F]fluciclovine to be optimal (Fig. 2). This is probably due to the bidirectional properties of the cellular transport system of leucine (29). While [^18^F]fluciclovine imaging for prostate cancers was performed almost immediately after injection *(30)*, the optimal time point for brain tumours is more uncertain and probably delayed (31). [^11^C]methionine PET for MM appears to usually be performed at 20 min p.i. (20, 22, 32), but we were unable to find reports investigating different timing. Here, the [^18^F]fluciclovine whole body images were obtained at 15 min p.i., but investigations of more immediate acquisitions could be interesting to pursue in a later study. The possibility of acquiring images earlier after administration of the radiopharmaceutical is nevertheless beneficial for several reasons. These include logistics for both patients and the nuclear medicine department as the time spent per patient is decreased. Furthermore, it will improve the image quality obtained for a certain absorbed dose, as early imaging allows decreased injected activity and/or acquisition time. Since the effective dose from the [^18^F]fluciclovine administration was already lower than for the [^18^F]FDG administration, we chose in our study to use a short acquisition time of 1 min per bed position, but this trade-off is open for optimisation.

The differences in visual appearance between [^18^F]fluciclovine and [^18^F]FDG PET were striking for many of the patients in the current study. While the IMPeTUs criteria can serve as guidance for the assessment of [^18^F]FDG PET (28), there are still many challenges associated with image interpretation. Only 9/13 patients were assessed positive on [^18^F]FDG PET in our study. Furthermore, the evaluations were, in several cases, not straightforward. The [^18^F]fluciclovine PET images were deemed positive in 12/13 patients and scored as easier to interpret than the according [^18^F]FDG PET scans for all positive cases. For the single subject assessed negative on both examinations, it should be noted that the [^18^F]FDG PET scan, unfortunately was performed 3 weeks before the [^18^F]fluciclovine scan. Theoretically, this subject could have turned [^18^F]FDG positive over the following weeks. For the rest of the population, the examinations were performed within 1 week of each other, in random order, and will most likely be highly comparable. One of the main limitations of the current work, besides the study size, is the lack of standardised assessment criteria for [^18^F]fluciclovine in MM. This is of course to be expected, as this pilot study is the first to explore the potential for this tracer. Still, especially pathological bone marrow uptake was challenging to characterise in a couple of cases, as normal physiological uptake of [^18^F]fluciclovine is expected in the marrow (30). This may also have a heterogeneous nature. Our institution has an extensive experience with [^18^F]fluciclovine for prostate cancer, and drew on this expertise for the current evaluations. While the majority of the [^18^F]fluciclovine images were easy to characterise, it should be noted that a few cases were borderline (especially subject 04). A set of evaluation criteria for MM, possibly also relaying on measurements of bone marrow heterogeneity and uptake structure, should be established if this tracer is to be used more routinely.

The approximate numbers of lesions were clearly different between the tracers (Table 2 and Fig. 1). While this number will not alter the positive/negative status, a study has reported a higher number of [^18^F]FDG foci to be associated with a less favourable prognosis (4), and an improved resolution of this number may therefore be relevant for stratification purposes. The semi-quantitative analyses for [^18^F]fluciclovine compared to [^18^F]FDG also support the visual results (Fig. 3) with significantly higher SUVs for [^18^F]fluciclovine vs [^18^F]FDG. Even though lesion selection for semi-quantitative measurements was done on both [^18^F]fluciclovine and [^18^F]FDG PET exams, choosing the lesions with highest uptake on each scan separately and then measuring the SUVs on both scans, all but one lesion had higher [^18^F]fluciclovine SUVs than [^18^F]FDG SUVs. This means that even the most visually striking lesions on [^18^F]FDG PET will probably have an even higher [^18^F]fluciclovine uptake. While no targeted biopsies were obtained to confirm that the lesions were indeed multiple myeloma, the follow-up [^18^F]fluciclovine examinations at 3 months after ASCT showed normalised uptake for all subjects. This, together with the improvement of the patients’ myeloma disease status, indicates that the lesions were actually myeloma. In this study, the mean SUVs for [^18^F]fluciclovine were more than twice as high as for [^18^F]FDG (8.2 vs 3.8, respectively). The ratios of the tumour to background tissue are also just as important for the assessments. Here, the tumour to blood ratio was more than three times higher for [^18^F]fluciclovine than [^18^F]FDG, and the tumour to bone marrow ratio was more than 40% higher for [^18^F]fluciclovine compared to [^18^F]FDG PET; further emphasising a beneficial [^18^F]fluciclovine uptake pattern. The liver is involved in the excretion of [^18^F]fluciclovine and, therefore, not a relevant tissue for thresholding. However, the ratio of tumour to liver for [^18^F]fluciclovine was still comparable, or even slightly higher, than that of [^18^F]FDG.

We here found a significant correlation between [^18^F]fluciclovine SUV measured in os ilium and the percentage of plasma cells in the biopsies, indicating that the PET examination represents a quantitative measure of tumour burden. No correlations were found for a similar investigation of [^18^F]FDG SUV. This is somewhat surprisingly, as earlier studies have found correlations also between biopsy results and [^18^F]FDG SUVs (33), but it may be that the study size in our investigation was too small to reveal a weak correlation for [^18^F]FDG. This is supported by an earlier study of [^18^F]FDG vs [^11^C]Methionine that found higher correlation coefficients for [^11^C]methionine PET (*r* = 0.6 for [^18^F]FDG vs *r* = 0.9 for [^11^C]methionine (33)). Although the biopsy data is from untargeted iliac crest marrow, introducing a potential uncertainty (especially for patients with patchy bone marrow pattern), this is no different from the previous study mentioned and should also not impact the correlation for each tracer differently.

Earlier studies have investigated alternative “non-[^18^F]FDG” tracers for MM, although also with a limited patient population, and in various clinical scenarios ranging from diagnosis to relapse. Since comparisons with [^18^F]FDG commonly has formed the basis for the investigations, direct evaluations between non-[^18^F]FDG tracers are challenging. While some tracers have shown somewhat disappointing results, such as [^18^F]FPRGD2 for integrin imaging (34) and Na[^18^F]F (9, 15), the investigation of others, such as [^11^C]acetate (14), seems to have been discontinued without apparent reasons. Carbon-11 or fluorine-18 labeled choline has been investigated in retrospective studies of relapse, progression, or for follow-up (18–20). While choline performed better than [^18^F]FDG (18, 19), it has later been shown inferior to [^11^C]Methionine is one of the rare studies comparing two non-[^18^F]FDG tracers (20). The amino acid-based [^11^C]Methionine has become an increasingly popular non-[^18^F]FDG tracer for MM and has, in several studies, shown higher sensitivity than [^18^F]FDG for detection of both intra- and extramedullary MM (21, 22, 33, 35). The combined evidence of [^11^C]methionine as a suitable MM tracer also holds promise for [^18^F]fluciclovine, as both tracers are amino acid-based, and the transporter system for [^11^C]Methionine (the L system) is also involved in cellular uptake of [^18^F]fluciclovine (24, 29). Our results support this theoretical notion and demonstrate that [^18^F]fluciclovine is indeed a promising tracer for MM. A recent study investigated [^11^C]methionine vs [^18^F]FDG in a study of 22 newly diagnosed, treatment-naïve symptomatic MM, finding that [^11^C]methionine is a more sensitive marker than [^18^F]FDG (22). While this study can be considered relatively comparable to ours, the high positive rate in both precludes speculations on which of the two amino acid-based tracers, [^11^C]methionine or [^18^F]fluciclovine, performs best. However, [^11^C]-Methionine is only feasible at centres with a cyclotron due to the short half-life of carbon-11. [^18^F]fluciclovine, being a fluorine-18-based tracer like [^18^F]FDG, is amenable to transport. Another amino acid-based tracer, [^18^F]fluoro-ethyl-tyrosine ([^18^F]FET), has also been investigated, although in a mixed population and only compared with CT imaging (36).

A potential disadvantage of [^18^F]fluciclovine and other amino acid-based tracers is the unavailability for theragnostic applications; replacing the positron emitter with a therapeutic nuclide. Tracers such as the CXCR4-targeting [^68^ Ga]Ga-pentixafor (13), PSMA tracers (currently under investigation), and the CD38-targeted daratumumab (16) have this potential. However, many theragnostic tracers, especially antibody-based, can be subject to downregulation. A broader selection of tracers, including [^18^F]fluciclovine or other amino acid-based tracers, should therefore be available to allow the best choice based on the clinical setting and stage.

## Conclusion

Based on this pilot study, [^18^F]fluciclovine is a promising PET tracer for MM patients. The visual assessments indicate that [^18^F]fluciclovine PET performs better then [^18^F]FDG PET at diagnosis, with both a higher number of active lesions present and improved interpretability of the bone marrow appearance. The semi-quantitative evaluations support this result, with higher SUVs, higher tumour to normal tissue values, and significant correlation with biopsy results observed only for [^18^F]fluciclovine SUVs. A similar study with a larger number of subjects should be performed to assert the results from this pilot.

## Supplementary Information

Below is the link to the electronic supplementary material.

Supplementary file1 (PPTX 1520 KB)
