# Supplementary material for: Comparison of [18F]fluciclovine and [18F]FDG PET/CT in Newly Diagnosed Multiple Myeloma Patients
Source: Mol Imaging Biol. 2022 May 2;24(5):842–51. doi: 10.1007/s11307-022-01734-0 (PMC9581841; doi:10.1007/s11307-022-01734-0)
Supplement: Supplementary file 1 — Supplementary file1 (PPTX 1520 KB) [file 11307_2022_1734_MOESM1_ESM.pptx]

## Slide 1
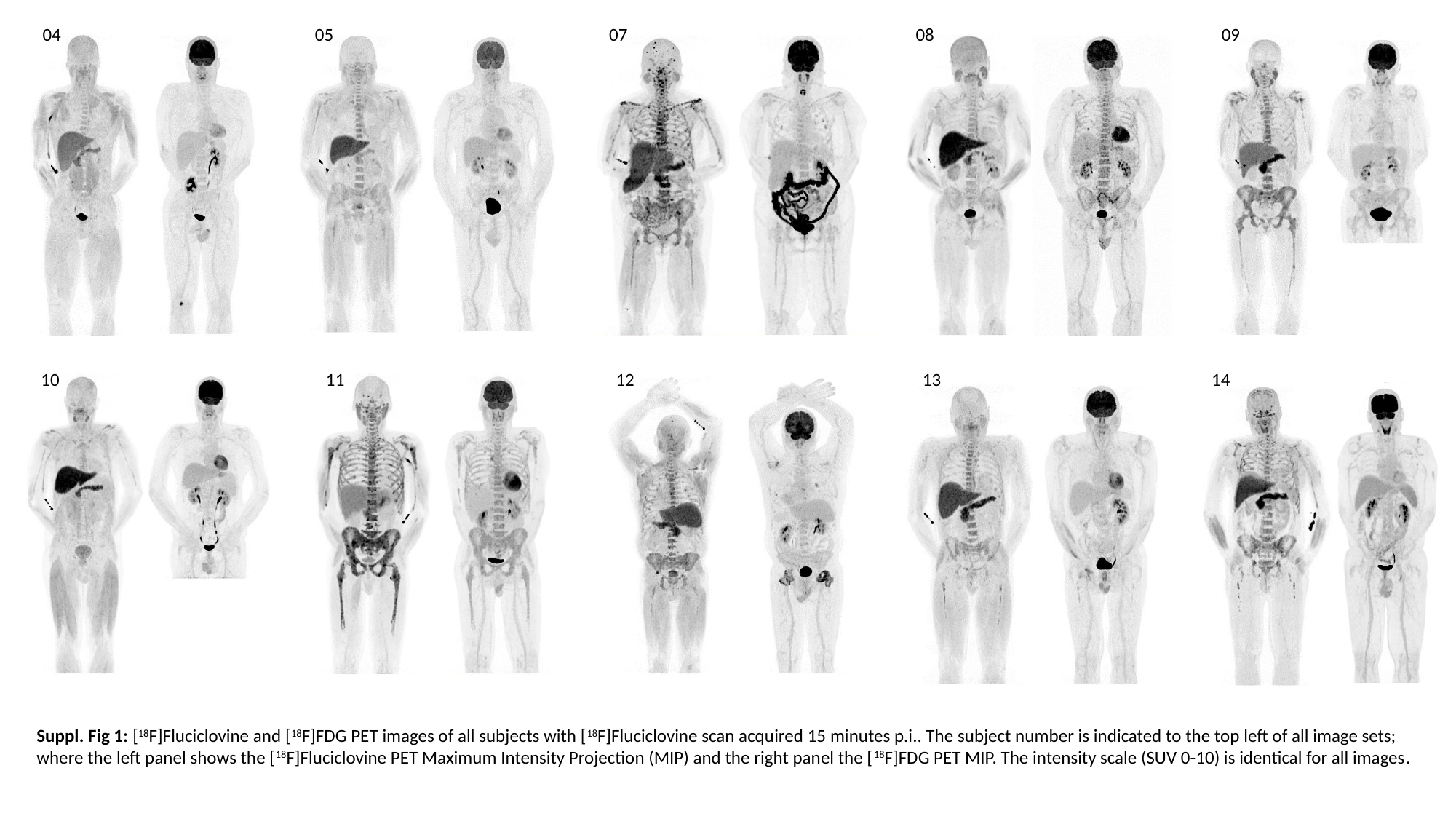

04		 05			 07			08		 09
10		 11			 12			 13		 14
Suppl. Fig 1: [18F]Fluciclovine and [18F]FDG PET images of all subjects with [18F]Fluciclovine scan acquired 15 minutes p.i.. The subject number is indicated to the top left of all image sets; where the left panel shows the [18F]Fluciclovine PET Maximum Intensity Projection (MIP) and the right panel the [18F]FDG PET MIP. The intensity scale (SUV 0-10) is identical for all images.
